# Invasive Fungal Infections in Children with Haematological Malignancies: Diagnostic and Therapeutic Challenges

**DOI:** 10.3390/jof7070516

**Published:** 2021-06-28

**Authors:** Athanasios Tragiannidis, Antonios Kattamis, Timoleon-Achilleas Vyzantiadis

**Affiliations:** 1 Children & Adolescent Haematology-Oncology Unit, Second Department of Paediatrics, Aristotle University of Thessaloniki, AHEPA Hospital, 53646 Thessaloniki, Greece; atragian@auth.gr; 2 Paediatric Haematology-Oncology Unit, First Department of Paediatrics, National and Kapodistrian University of Athens, Aghia Sophia Children’s Hospital, 11527 Athens, Greece; ankatt@med.uoa.gr; 3 First Department of Microbiology, Medical School, Aristotle University of Thessaloniki, 54124 Thessaloniki, Greece

The incidence of invasive fungal infections (IFIs) has dramatically increased over the last few decades in parallel with the increased number of immunocompromised patients. Children with haematological malignancies and those undergoing haematopoietic stem cell transplantation (HSCT) are at risk of developing invasive fungal infections [1]. Despite improvements in diagnostic tools and therapeutic options, the morbidity and mortality related with invasive mycoses in children remains high [1,2,3,4,5,6].

Timely diagnosis and prompt initiation of antifungal treatment are essential for improving the outcome. Various international guidelines have been proposed for the diagnosis, prevention, and treatment of invasive mycoses in children. Even though there has been an improvement in diagnostic assays, their role in establishing the diagnosis of IFIs has not been regularly assessed in pediatric populations. In this respect, pediatric data and pediatric recommendations for some of these diagnostic tests are either still completely lacking or are focused on specific pediatric populations, such as neonates.

In children, the standard diagnostic procedures for IFIs, while not supported yet by strong recommendations, do not differ from those of adults. The main target is always the early detection of the possible pathogen and the consequent application of therapy, as well as the monitoring of the clinical condition. Apart from of the usefulness of a fungal biomarker such as mannan, galactomannan or β-D-glucan, or a molecular approach such as PCR for *Aspergillus* or *Candida*, there are specific difficulties in sampling, both culture and microscopy, which should always be attempted prior to the initiation of the antifungal treatment. Of course, as innovative methodologies are validated and information is arising in relation to specific fungal pathogens and groups of pediatric patients, the more all of these laboratory approaches are able to contribute to diagnosis and targeted treatment [1,7].

Three classes of drugs are mainly used for the management of invasive mycoses in children: older and newer azoles, polyenes, and echinocandins. Each have different mechanisms of action, activity, and susceptibility. Pediatric patients at high risk for the development of invasive mycoses include patients with acute myeloid leukemia, recurrent and/or refractory acute lymphoblastic leukemia (ALL), high risk ALL, and those who undergo HSCT [1]. While different guidelines have been proposed, they all recommend the use of antifungal prophylaxis, based mainly on the widespread use of azoles such as fluconazole and Posaconazole, and, less frequently, other azoles (itraconazole, voriconazole), echinocandins, and liposomal amphotericin B (LAmB) [1]. Empirical antifungal therapy (fever-driven) focuses on children with febrile neutropenia after 96 h of unresponsiveness to empiric broad-spectrum antibiotic therapy. Liposomal amphotericin B and caspofungin are both approved for empiric antifungal treatment in children without an age restriction (Grade I recommendation, level of evidence I) [1]. A pre-emptive or diagnostic-driven approach is based on the use of clinical, microbiological, and radiological criteria in neutropenic pediatric patients, which seeks to reduce the use of unnecessary antifungal treatment, as shown by comparative studies [8]. This approach is currently recommended among pediatric patients (grade B recommendation, level of evidence II). Guidelines for targeted primary therapy for invasive candidiasis/candidaemia and invasive aspergillosis recommend the use of echinocandins and voriconazole, respectively [1,9,10]. Treatment recommendations for mucormycosis in children favor the prompt initiation of high doses of liposomal amphotericin B and surgery [1]. Finally, the new era of precision medicine in the field of pediatric hematology oncology has shown promising results through the use of targeted therapies. Surveillance and thorough evaluation for IFIs is needed, especially for children on chemotherapy who are immunocompromised [11].

In our view, the broader application of existing diagnostic methodologies offers, in short time, all the necessary data for their full validation and evaluation in pediatric populations. In this context, broad clinical mycology networks at the national or regional level comprising specialized clinical and laboratory departments will greatly facilitate the use of existing methodologies, the referral of specimens, and will offer the necessary diagnostic coverage to all patients.

This Special Issue focuses on the latest research and development of diagnostic tools and newer antifungals in children with hematological malignancies.
